# The aminotransferase Aat initiates 3-phenyllactic acid biosynthesis in *Pediococcus acidilactici*

**DOI:** 10.3389/fmicb.2023.1150425

**Published:** 2023-04-28

**Authors:** Alexander Wenger, Cornelia Bär, Reto Portmann, Remo S. Schmidt, Elisabeth Eugster, Laure Weisskopf, Stefan Irmler

**Affiliations:** ^1^Agroscope, Bern, Switzerland; ^2^Department of Biology, University of Fribourg, Fribourg, Switzerland; ^3^School of Agricultural, Forest and Food Sciences HAFL, Bern University of Applied Sciences, Zollikofen, Switzerland

**Keywords:** *Pediococcus acidilactici*, aminotransferase, knockout, 3-phenyllactic acid, 4-hydroxyphenyllactic acid

## Abstract

The function of the aminotransferase Aat (GenBank Protein WP_159211138) from *Pediococcus acidilactici* FAM 18098 was studied *in vivo*. For this purpose, the gene was replaced with an erythromycin resistance gene using the temperature-sensitive *Escherichia coli-Pediococcus* shuttle plasmid pSET4T_Δ*aat*. The knockout was verified by PCR and genome sequencing. Subsequently, the differences between the metabolism of the knockout and of the wild-type strain were investigated by determining the free amino acids and organic acids in culture supernatants. It was found that the knockout mutant no longer synthesized 3-phenyllactic acid (PLA) and 4-hydroxyphenyllactic acid (HPLA). Additionally, the mutant strain no longer catabolized phenylalanine. Metabolic pathway analysis using the KEGG database indicate that *P. acidilactici* cannot synthesize α-ketoglutarate that is a predominant amino-group acceptor in many transamination reactions. To study the transfer of the amino group of phenylalanine, the wild-type strain was incubated with [^15^N] phenylalanine. Mass spectrometry showed that during fermentation, [^15^N] alanine was formed, indicating that pyruvic acid is an amino group acceptor in *P. acidilactici*. The present study shows that Aat plays a crucial role in PLA/HPLA biosynthesis and pyruvic acid is an amino acceptor in transamination reactions in *P. acidilactici*.

## 1. Introduction

Fermentation has been used for hundreds of years to preserve food. Many fermentations rely on the metabolic activity of lactic acid bacteria. These bacteria form lactic acid from sugar and thus lower the pH of the food, resulting in a longer shelf life. Nowadays, when lactic acid bacteria are selected for fermentation, it is not only important that they perform a fast acidification but also that they possess specific properties. These include, for example, the formation of aroma substances and/or antimicrobial substances.

Starter cultures are used for controlled acidification during cheese manufacture. During cheese ripening, non-starter lactic acid bacteria then start to grow (Beresford and Williams, 2004). They are a significant component of the cheese microbiome at the end of ripening and can affect cheese quality both positively and negatively. Amongst others, *Pediococcus acidilactici* can be part of the non-starter lactic acid bacteria population.

We are interested in the amino acid metabolism of *P. acidilactici* since it exhibits properties that can influence cheese quality. For example, it has been reported that it can produce 3-phenyllactic acid (PLA) and 4-hydroxyphenyllactic acid (HPLA) from phenylalanine and tyrosine, respectively (Mu et al., 2012b). Both compounds, PLA and HPLA, exhibit antifungal activity (Lavermicocca et al., 2000). Additionally, it possesses the genes of the arginine deiminase pathway, which converts arginine to ornithine (Eugster et al., 2019; Wenger et al., 2020). This metabolic pathway releases carbon dioxide which could influence the eye formation in cheese. Finally, *P. acidilactici* can degrade serine and threonine and, in turn, form alanine and α-aminobutyric acid in medium and cheese (Eugster et al., 2019; Wenger et al., 2020). We assume that serine and threonine are converted to alanine and α-aminobutyric acid, respectively. Our hypothesis is that a dehydratase metabolizes serine and threonine to α-keto acids, which are substrates for both aminotransferases and dehydrogenases.

When we searched the genome sequence of *P. acidilactici* strain FAM 18098 for genes encoding aminotransferases, an aminotransferase class I/II-fold pyridoxal phosphate-dependent enzyme (GenBank Protein WP_159211138) was identified that may be involved in the formation of α-aminobutyric acid and alanine (Wenger et al., 2020). In order to analyze the enzymatic activity of this aminotransferase, the gene was cloned and overexpressed in *Escherichia coli.* We found that the purified aminotransferase exhibited activity toward various amino acids such as leucine, methionine, α-aminobutyric acid, alanine, cysteine, and phenylalanine. Because of the broad substrate specificity, we could not clearly deduce whether the aminotransferase plays a role in the formation α-aminobutyric acid or alanine *in vivo*.

The aim of this work was to study the role of aminotransferase Aat *in vivo*. For this purpose, the gene was inactivated in FAM 18098 by gene replacement. The composition of the organic acids and amino acid in the culture supernatants of the knockout strain was compared with those of the wild-type strain. When it became apparent that the mutant strain no longer synthesized PLA from phenylalanine, further studies were performed with [^15^N]-labeled phenylalanine to identify the *in vivo* amino group acceptors.

## 2. Materials and methods

### 2.1. Bacterial strains, media, and growth conditions

The bacterial strains used in this study are listed in Table 1. *P. acidilactici* strains were cultivated in MRS medium (de Man et al., 1960) at 30°C.

**Table 1:** Strains and plasmids used in this study.

Strain/Plasmid	Genotype, relevant features	Source
*E. coli*		
TOP10	F-*mcrA* Δ(*mrr-hsd*RMS-*mcr*BC) Φ80*lac*ZΔM15 Δ *lac*X74 *rec*A1 *ara*D139 Δ(*araleu*)7697 *gal*U *gal*K *rps*L (StrR) *end*A1 *nup*G	Invitrogen
TOP10/pUC19_*tetM*	TOP10 harboring pUC19_*tetM*	This study
TOP10/pUC19_*ermA*	TOP10 harboring pUC19_*ermA*	This study
TOP10/pSET4T	TOP10 harboring pSET4T	This study
TOP10/pSET4T_∆*aat*	TOP10 harboring pSET4T_∆*aat*	This study
*P. acidilactici*		
FAM 18098	Cheese isolate	Agroscope culture collection
FAM 13875	Cheese isolate, Em^R^, Tet^R^	Agroscope culture collection
FAM 18098/pSET4T_∆*aat*	FAM 18098 harboring pSET4T_∆*aat*	This study
FAM 26181	FAM 18098 *aat* deficient strain, Em^R^	This study
Plasmids		
pUC19	Amp^R^	Invitrogen
pUC19_*tetM*	*tet(M)* with native promoter from FAM 13875 cloned in pUC19	This study
pUC19_*ermA*	*erm(A)* with native promoter from FAM 13875 cloned in pUC19	This study
pSET4s	Temperature-sensitive vector, ColE1, repAts, Spc^R^	Nova Lifetech Limited
pSET4T	Temperature-sensitive vector, ColE1, repAts, Tet^R^	This study
pSET4T_∆*aat*	*aat* knockout plasmid, Tet^R^, Em^R^	This study

Amp^R^, ampillicin resistance; Spc^R^, spectinomycin resistance; Tet^R^, tetracycline resistance; Em^R^, erythromycin resistance.

To study amino acid metabolism, strains were grown at 30°C for 4 days in basal medium (pH 7.0 ± 0.2) that consisted of di-potassium hydrogen phosphate anhydrous (9 g L^−1^), yeast extract (5 g L^−1^), casein hydrolysate (2 g L^−1^), magnesium sulfate heptahydrate (0.2 g L^−1^), manganese dichloride tetrahydrate (0.2 g L^−1^), D-galactose (2 g L^−1^), 5 mM of L-serine, and 5 mM of L-threonine.

To study the transfer of the amino group of phenylalanine, *P. acidilactici* strain FAM 18098 was grown at 30°C for 3 days in MRS medium supplemented with 5 mM of [^15^N] L-phenylalanine (Cambridge Isotope Laboratories, Tewksbury, MA, United States).

*Escherichia coli* strains were grown in LB medium (Sambrook et al., 1989), with shaking (220 rpm) at 37°C. The LB medium was supplemented with ampicillin (100 μg mL^−1^) or tetracycline (50 μg mL^−1^), if necessary.

### 2.2. General molecular biology techniques

Genomic DNA (gDNA) was extracted as described by Berthoud et al. (2017), and DNA concentration was determined using the Qubit dsDNA BR Assay Kit (Thermo Fisher Scientific, Reinach, Switzerland). Plasmids were isolated using the FastGene Plasmid Mini Kit (Nippon Genetics Europe, Düren, Germany). Restriction and ligation were performed using the Invitrogen Anza Restriction Enzyme Cloning System (Thermo Fisher Scientific). One Shot TOP10 Chemically Competent *E. coli* (Thermo Fisher Scientific) were used for the transformation of *E. coli.*

The primers were designed using the Primer3Plus tool (Untergasser et al., 2012) and synthesized by Microsynth AG (Balgach, Switzerland). Their sequences are listed in Table 2. The polymerase chain reactions (PCR) were performed using the Phusion High-Fidelity DNA Polymerase (Thermo Fisher Scientific) following the manufacturer’s instructions and used in all PCR reactions. The amplicons were purified using the FastGene Gel/PCR Extraction Kit (Nippon Genetics Europe). Their concentrations were determined using a NanoDrop Spectrophotometer (Thermo Fisher Scientific).

**Table 2:** Primers used in this study.

Primer	Sequence 5′ to 3′	Binding site	Utilization
P1	ATATGGATCCGCTCCTAAAAGATGGGTTTGTG	*tet(M)*	cloning in pUC19
P2	ATATGAATTCGGCAACCCAAATCTCGCAAT	*tet(M)*	cloning in pUC19
P3	AGAAAACGTCCAACTGAAATGTAAAGTG	*erm(A)*	cloning in pUC19 & creating pSET4T_Δ*aat*
P4	TTAGGAAGAATAAAGCGTTCTCTTGTG	*erm(A)*	cloning in pUC19 & creating pSET4T_Δ*aat*
P5	TTACTAGTGCTCCTAAAAGATGGGTTTGTG	*tet(M)*	creating pSET4T
P6	TTACTAGTGGCAACCCAAATCTCGCAAT	*tet(M)*	creating pSET4T
P7	gcgggtgttggcgggtgtcggggctggcttaactatgcggCGTTCGCCAAATCCTCCA	downstream of *aat*	creating pSET4T_Δ*aat*
P8	tgtaatttttgcactttacatttcagttggacgttttctaCCTCAAAGCTAAACGAGCA	*aat*	creating pSET4T_Δ*aat*
P9	cgttctctaatttcacaagagaacgctttattcttcctaaGTGCAGTCCTCCTCATATTAAAATGG	upstream of *aat*	creating pSET4T_Δ*aat*
P10	aacaatttcacacaggaaacagctatgaccatgattacgcCAATCCCAGCGTAAATAATAGTAGGA	upstream of *aat*	creating pSET4T_Δ*aat*
P11	GCGTAATCATGGTCATAGCTGT	pSET4T	creating pSET4T_Δ*aat*
P12	CCACGTAATATCGTAGAAGCGGA	pSET4T	creating pSET4T_Δ*aat*
P13	TGATGCCCTTTTGGAAATCTCAG	pSET4T	creating pSET4T_Δ*aat*
P14	CCGCATAGTTAAGCCAGCCC	pSET4T	creating pSET4T_Δ*aat*
P15	TTTTTCAATCCGCGCAATTGCGTT	*aat*	screening for the gene replacement
P16	AACGCACTATTAGAACGCATCAAACCA	*aat*	screening for the gene replacement
P17	CGCACGTTCGCCAAATCCTCCAAG	downstream of *aat*	screening for the gene replacement
P18	AGCGGTGGTAGTTCTGATAGGCAT	upstream of *aat*	screening for the gene replacement
P19	AATGACGGGCAAATCGGTCGAAC	downstream of *aat*	screening for the gene replacement
P20	TCAATAGCAAACCTAAAGCTCGTTTCGT	*erm(A)*	screening for the gene replacement
P21	GTCAAAATGAGTCGGTGGGTGGAT	*erm(A)*	screening for the gene replacement
P22	CCAGTTCGGCTTGGCTAAGGTC	upstream of *aat*	screening for the gene replacement

Restriction sites are underlined; overlaps needed for Gibson Assembly are indicated in lowercase.

### 2.3. Construction of the *aat*-gene knockout plasmid pSET4T_∆*aat*

**Figure 1:**
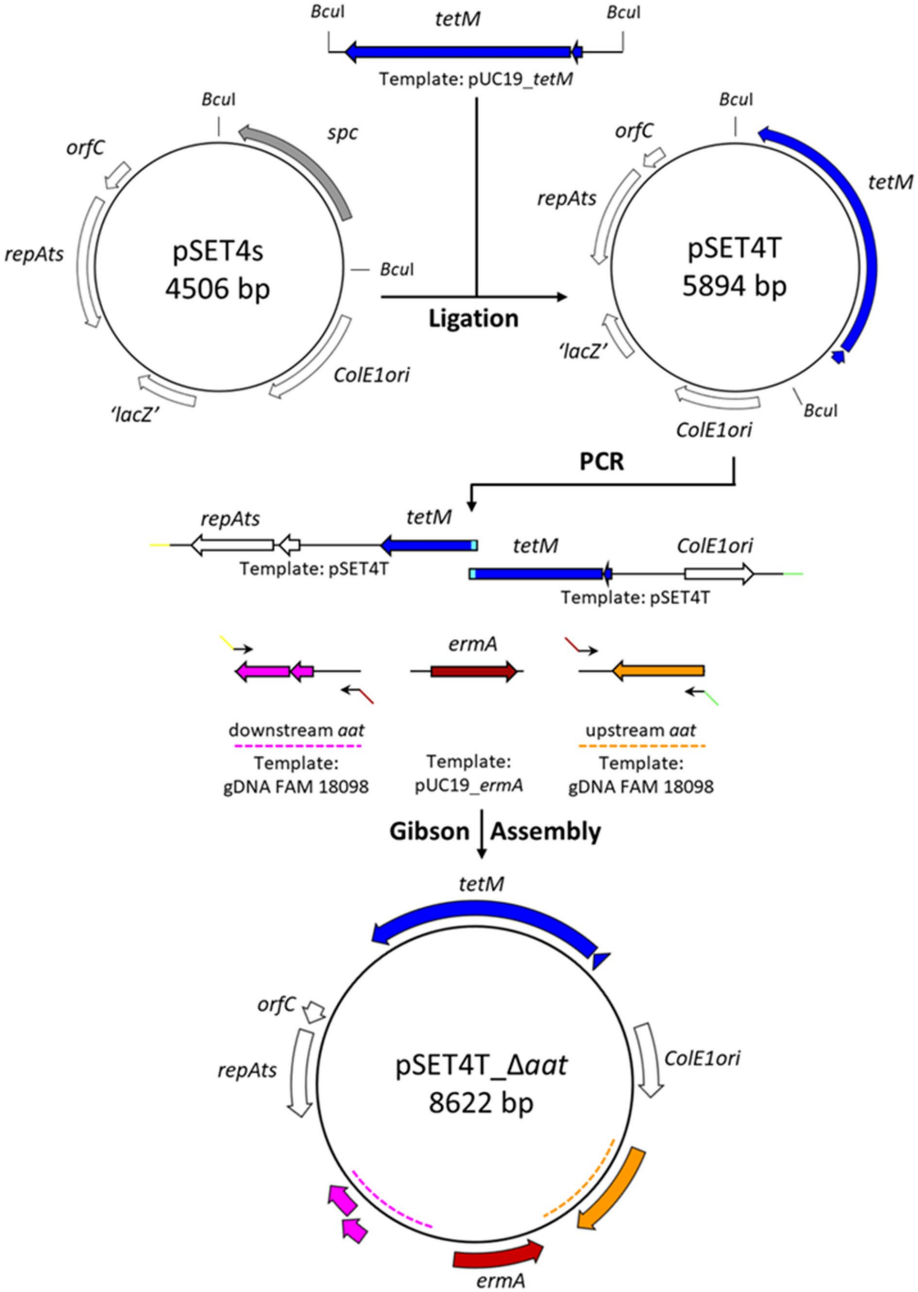
Schematic representation of the construction of the gene knockout plasmid pSET4T_*Δaat*. Tetracycline resistance gene *tetM* was amplified by PCR from pUC19_*tetM* vector and ligated into pSET4S using the two *Bcu*I sites creating pSET4T. The gene replacement vector pSET4T_*Δaat* was constructed by PCR amplification of the plasmid backbone of pSET4T with two overlapping fragments (indicated in light blue). The regions 1-kbp upstream and 1-kbp downstream of *aat* were amplified from the genomic DNA of *P. acidilactici* strain FAM 18098 using overhang primers with homologous sequences of the other fragments (indicated by colored arrows). The erythromycin resistance gene *ermA* was amplified via PCR from pUC19_*ermA*. The five fragments were assembled using a Gibson Assembly resulting in pSET4T_*Δaat*. The wide arrows indicate open reading frames.

The workflow for the construction of pSET4T_∆*aat* is shown in Figure 1. The gene *tet(M)* (GenBank Nucleotide GBP49_00310) including the promoter region was amplified from the gDNA of *P. acidilactici* strain FAM 13875 using primers P1/P2. The amplicon was purified then digested with *Bam*HI and *Eco*RI and ligated in the *Bam*HI- and *Eco*RI-digested pUC19, resulting in pUC19_*tetM*. *E. coli* TOP10 were then transformed with the ligation reaction, and transformants were selected on LB agar containing tetracycline.

The gene *erm(A)* (GenBank Nucleotide GBP49_00290) including the promotor region was amplified from the gDNA of *P. acidilactici* strain FAM 13875 using primers P3/P4 and ligated to *Sma*I-digested pUC19. The ligation reaction was transformed into *E. coli* TOP10, which were then plated on LB agar supplemented with ampicillin and X-Gal. Transformants were selected by blue/white screening. Sanger sequencing was used to check the orientation of the insert.

Tetracycline resistance gene *tet(M)* was amplified from the pUC19_*tetM* plasmid using the primers P5/P6. The amplicon was digested by *Bcu*I and then ligated into *Bcu*I-digested pSET4S. After transformation into *E. coli* TOP10, transformants were selected on LB agar containing tetracycline. The resulting plasmid was named pSET4T.

The final gene replacement vector pSET4T_∆*aat* was created using a Gibson Assembly reaction. Therefore, the 1-kbp upstream and downstream regions of *aat* were amplified using the primers P7/P8 and P9/P10, respectively, from the gDNA of strain FAM 18098. The *erm(A)* gene was amplified with the primers P3/P4 from pUC19_*ermA*. Furthermore, two fragments of pSET4T were amplified using the primers P11/P12 and P13/P14. The five amplicons were purified and then assembled using the GeneArt^™^ Gibson Assembly^®^ HiFi Cloning Kit (Thermo Fisher Scientific, Reinach, Switzerland) following the manufacturer’s instructions. The reaction mix was transformed into *E. coli* TOP10, and transformants were selected on LB agar containing tetracycline. The composition of the plasmids isolated from several transformants was analyzed by restriction analysis and PCR combined with Sanger sequencing. A correctly assembled plasmid was named pSET4T_∆*aat* and propagated and maintained in *E. coli* TOP10.

### 2.4. Engineering of an *aat* knockout mutant strain

Using the protocol described by Caldwell et al. (1996), pSET4T_∆*aat* (220 ng) was transformed into *P. acidilactici* FAM 18098 by electroporation. Transformants were selected on MRS plates supplemented with erythromycin (5 μg mL^−1^) and grown at 30°C for 48 h. A transformant colony was then cultured in MRS medium containing erythromycin at 42°C to stop plasmid replication. After 24 h of growth, transformants were reinoculated into fresh MRS medium supplemented with erythromycin, creating a new culture. Colonies were obtained by diluting a sample of each culture by a factor of 10^−6^ and 10^−7^ with NA (0.8% sodium chloride, 0.1% peptone from casein, pancreatic digest), which was then plated on MRS agar containing erythromycin. Each time, 50 colonies were transferred with a sterile toothpick onto a new MRS agar plate containing erythromycin. After incubating for 24–48 h, colonies were replica plated onto an MRS agar plate containing tetracycline (100 μg mL^−1^). Colonies exhibiting resistance against erythromycin and sensitivity toward tetracycline were analyzed for the loss of pSET4T_∆*aat* and the replacement of *aat* using PCR with the primers P15–P22 (Table 2).

The gDNA of a mutant of interest (strain FAM 26181) was sequenced using Illumina Novaseq technology at the Next Generation Sequencing Platform of the University of Bern. The Illumina reads were trimmed using Trimmomatic (version 0.38, options: SLIDINGWINDOW:4:8 MINLEN:127) (Bolger et al., 2014). The trimmed reads were mapped to the genome sequence of *Pediococcus acidilactici* FAM 18098 (GenBank Assembly ASM980809v1) using bowtie2 (Langmead and Salzberg, 2012). Samtools (version 1.16.1) (Danecek et al., 2021) was then used to sort and index the reads and to identify regions with zero coverage. Furthermore, the trimmed reads were assembled using SPAdes (version 3.6.1, options: -careful -mismatch-correction -k 21,33,55,77,99,127). The assembly was deposited in the GenBank database under the accession number ASM2453963v1.

### 2.5. Determination of free amino acids and organic acids

Free amino acids in culture supernatants were determined using high-performance liquid chromatography (HPLC) as previously described (Wenger et al., 2020).

### 2.6. Determination of organic acids

Organic acids were determined by HPLC using an Aminex HPX-87H column (Bio-Rad, Cressier, Switzerland). Perchloric acid was added to the culture supernatant at a final concentration of 0.5 M, and the supernatant was then filtered (Nylon 0.45 μm, Millipore Millex-HN, Merck KGaA, Darmstadt, Germany). The chromatographic conditions were as follows: column temperature = 65°C, flow rate = 0.4 mL min^−1^, UV detection at 210 nm, injection volume = 2 μL. Organic acids, such as oxalic acid, citric acid, malic acid, succinic acid, formic acid, acetic acid, pyruvic acid, lactic acid, α-ketoglutaric acid, α-ketobutyric acid, phenyllactic acid, and 4-hydroxyphenyllactic acid, were used as standards.

### 2.7. LC–MS analyses

Analyses were performed using a Vanquish liquid chromatography system coupled to a Q Exactive Plus Orbitrap mass spectrometer (Thermo Fisher Scientific) and controlled by the Thermo Xcalibur software (version 4.5.474.0, Thermo Fisher Scientific). Samples were filtered before analysis (nylon, 0.45 μm, Millipore Millex-HN, Merck KGaA, Darmstadt, Germany) and then separated on an Acquity UPLC HSS T3 column (150 × 2.1 mm i.d., 1.8 μm particle size; Waters Corporation, Milford, MA, United States) at a flow rate of 400 μL min^−1^ at 40°C. Mobile phase A consisted of H_2_O containing 0.1% formic acid, and mobile phase B consisted of acetonitrile containing 0.1% formic acid. Mass spectra were recorded at a resolution of 140,000 FWHM (full width at half maximum). Peak areas were determined using the Thermo Xcalibur Qual Browser software (version 4.1.31.9, Thermo Fisher Scientific).

For the determination of PLA and HPLA, 10 μL of the sample was injected. The gradient was as follows: 5% B for 2 min; 5–95% B for 17 min; 95% B for 4 min. Mass spectra were recorded over a mass range of 66.7–300 in negative ionization mode. The mass ranges from 165.045 to 165.065 m/z and from 181.045 to 181.065 m/z were used to analyze extracted ion chromatograms for PLA and HPLA, respectively. The results shown in this study reflect the mean and standard deviation of three independently performed biological experiments.

For the detection of [^15^N]-labeled amino acids, a standard solution containing the amino acids alanine, aspartic acid, glutamic acid, leucine, methionine, phenylalanine, valine, and 2-aminobutyric acid (each 1 pmol μL^−1^) was measured in triplicate. The measurements were used to determine retention times and to verify the accurate masses of the monoisotopic [^14^N]-peaks and the masses of the first naturally occurring [^15^N]-containing isotope of the amino acids. Filtered samples with or without incubation of *Pediococcus acidilactici* FAM 18098 were diluted hundredfold with water, of which 1 μL was injected and separated using the following gradient: 3% B for 3 min; 3–95% B for 7 min; 95% B for 5 min. Mass spectra of the samples were recorded over a mass range of 75–300 in positive ion mode. Extracted ion chromatograms of the monoisotopic [^14^N]- and the [^15^N]-peak with a tolerance of 2 ppm were generated to determine the peak areas for the substances of interest (Supplementary Table S1).

## 3. Results

### 3.1. Construction of an *aat* knockout mutant

In order to better understand the function of *aat*, the gene was knocked out in this study using the thermosensitive vector pET4s, which was originally constructed for gene replacements in *Streptococcus suis* and carries the *spc* gene that confers spectinomycin resistance (Takamatsu et al., 2001). Because *P. acidilactici* FAM 18098 is resistant to spectinomycin, we selected tetracycline and erythromycin resistance genes as markers of selection. For this purpose, the *tet(M)* and *erm(A)* gene of *P. acidilactici* FAM 13875, a strain that showed resistance to tetracycline and erythromycin (Lüdin et al., 2018), were cloned. The final plasmid pSET4T_Δaat consisted of two origins of replication (ColE1ori and orfC/repAts), the *tet(M)* gene, and the nucleotide sequences needed for the exchange of the *aat* gene with *erm(A)* (Figure 1).

After transformation of strain FAM 18098 with the knockout plasmid, transformed cells were selected with erythromycin. Initially, the colonies also showed resistance to tetracycline. After three cultivation steps, a tetracycline-sensitive colony was found. PCR analysis confirmed that the colony had lost both the knockout plasmid and the *aat* gene (data not shown). The culture obtained from this colony was named FAM 26181.

**Figure 2:**
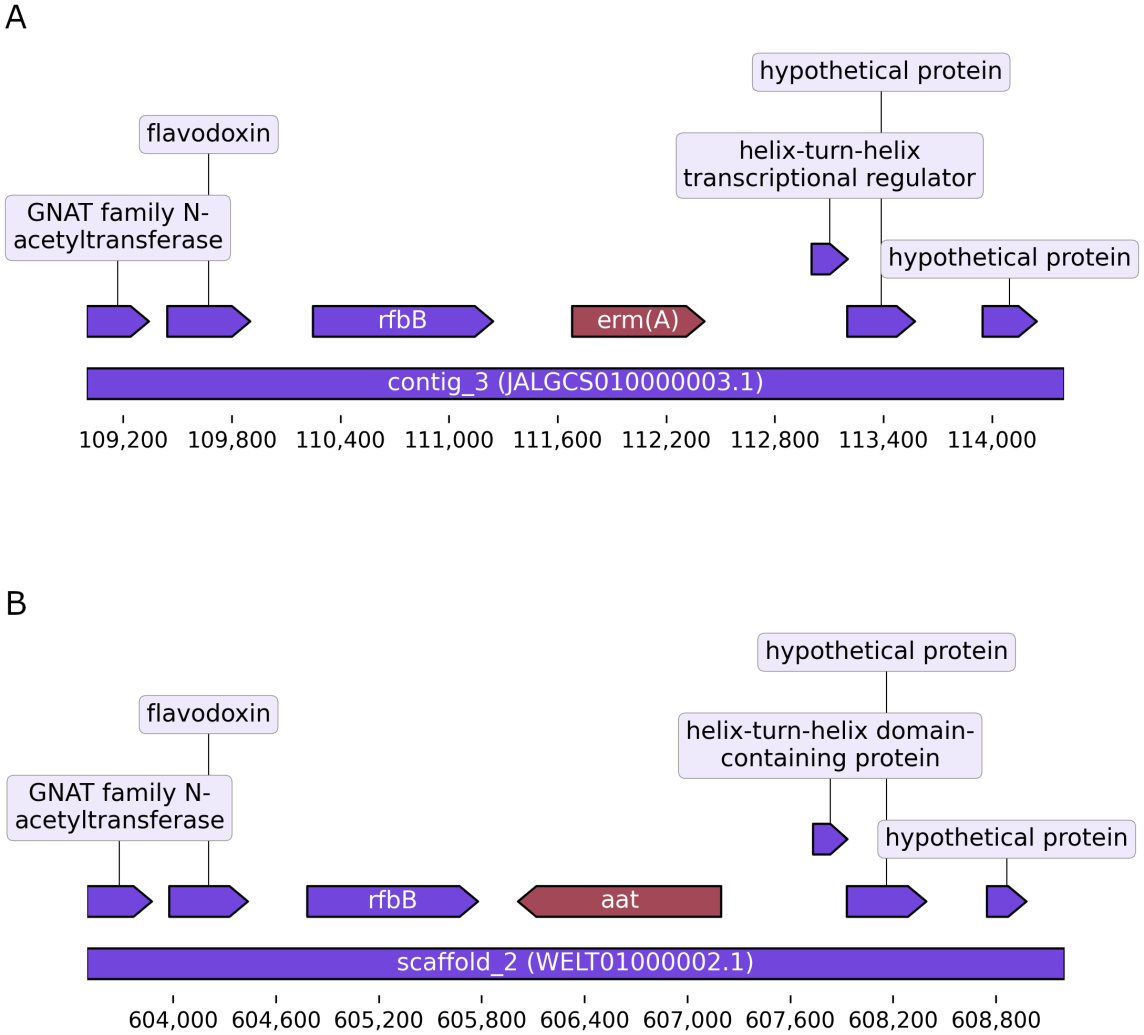
Gene replacement in *Pediococcus acidilactici.* The illustration shows the *erm(A)* gene that replaced the *aat* gene in the knockout strain **(A)**. The corresponding region containing the *aat* gene in the wild-type strain is shown in the lower panel **(B)**. Arrows represent protein-coding sequences. The rulers indicate the location in contig_3 (GenBank acc. no. JALGCS010000003.1) and chromosome (GenBank acc. no. WELT01000002.1).

In order to validate the disruption of the *aat* gene, the gDNA of this strain was sequenced using Illumina sequencing and compared with the already existing genome sequence of the wild-type strain FAM 18098. When the Illumina reads obtained from the knockout strain were mapped to the genome assembly of the wild-type, the region from position 606,059 to 607,168 of the wild-type’s chromosome containing the *aat* gene was not covered (data not shown). This confirmed the results of the PCR analysis and showed that the *aat* gene was no longer present in the knockout strain. The analysis of the *de novo* assembly showed that the *erm(A)* gene had been integrated at this site (Figure 2).

### 3.2. Free amino acid composition in culture supernatants

Since the formation of α-aminobutyric acid by *P. acidilactici* can be observed in a so-called basal medium (Wenger et al., 2020), this medium was used to compare the amino acid metabolism of the *aat* knockout mutant with that of the wild-type strain.

**Figure 3:**
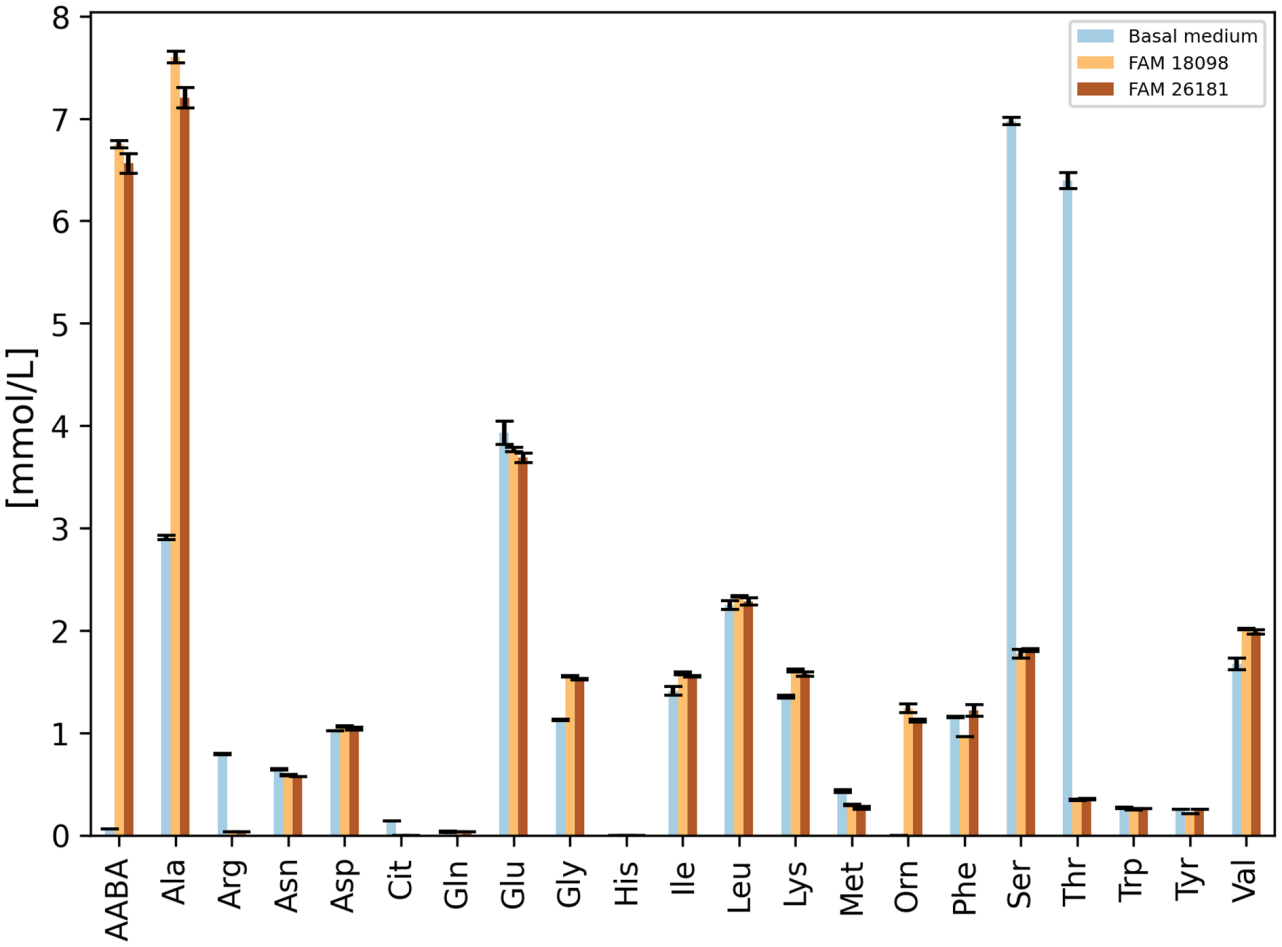
Amino acid concentrations in culture supernatants of wild-type strain FAM 18098 and *aat* knockout strain FAM 26181 after incubation for 4 days in the basal medium. Values represent the mean (±standard deviation) of two independent biological experiments. AABA: α-aminobutyric acid, Cit: citrulline, Orn: ornithine. All other amino acids are abbreviated according to IUPAC nomenclature.

When amino acid levels were determined in culture supernatants, the knockout strain was found to produce α-aminobutyric acid and alanine and to degrade serine and threonine (Figure 3). This was also true for the wild-type strain, except that the levels of α-aminobutyric acid and alanine were slightly higher than in the culture supernatant of the knockout strain. Furthermore, it was observed that both strains metabolized arginine and formed ornithine.

It was noticeable that the phenylalanine content in the culture supernatants of the wild-type strain was considerably lower than in the basal medium and in the culture supernatants of the knockout strain (Figure 3). A similar, but less pronounced pattern was observed for tyrosine.

### 3.3. Determination of PLA and HPLA in culture supernatant

Since various lactic acid bacteria are able to form PLA in MRS medium (Mu et al., 2012c), this medium was used to compare the organic acid profile of the wild-type strain with that of the knockout strain.

**Figure 4:**
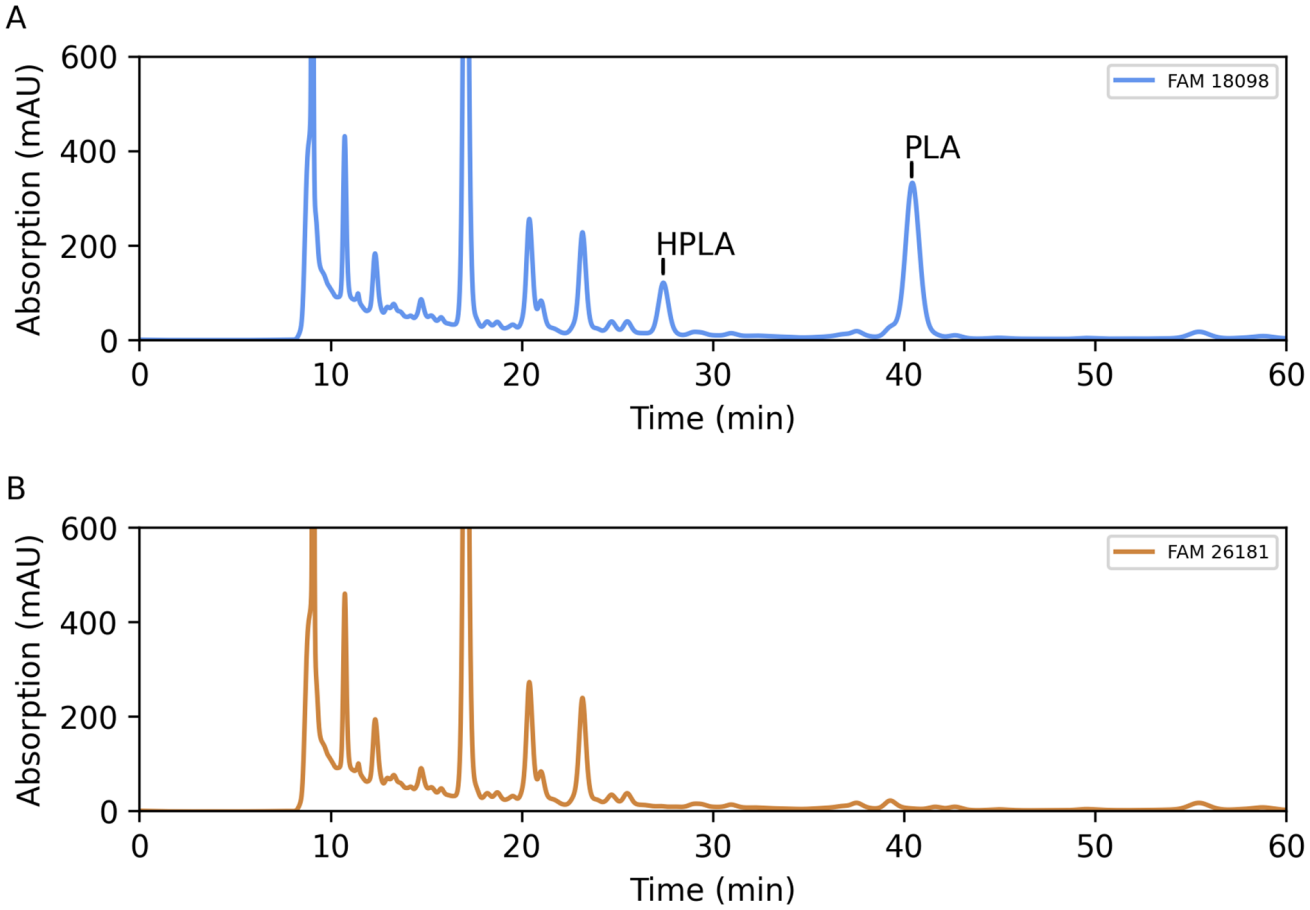
Organic acid profile of culture supernatants of wild-type strain FAM 18098 **(A)** and of *aat* knockout strain FAM 26181 **(B)** after incubation for 3 days in MRS medium. PLA: 3-phenyllactic acid, HPLA: 4-hydroxyphenyllactic acid.

Comparison of the organic acid chromatograms showed that two peaks were missing in the culture supernatant of the knockout strain (Figure 4). The first peak eluted with a retention time consistent with that of HPLA. The retention time of the second peak was consistent with that of PLA.

In order to verify the identity of the two substances, a mass spectrometry method was established to specifically and quantitatively determine PLA and HPLA in the culture supernatants. Using standard curves generated with the pure substances, it was found that wild-type strain FAM 18098 produced 1.19 ± 0.11 mmol/L PLA and 0.38 ± 0.04 mmol/L HPLA in MRS medium, while neither substance was detected in the medium or in the culture supernatants of FAM 26181.

### 3.4. Studies on the transfer of the nitrogen group of phenylalanine

Since wild-type strain catabolizes phenylalanine, as described above, we investigated whether the amino group of phenylalanine is transferred to an α-keto acid. This would indicate that an aminotransferase is involved in the degradation of phenylalanine. For this purposse, [^15^N]-labeled phenylalanine was added to the medium and fermented with *P. acidilactici* FAM18098.

The following amino group acceptors were considered: pyruvic acid, α-ketobutyric acid, oxaloacetic acid, α-ketoglutaric acid, α-ketoisocaproic acid, α-ketomethylthiobutyric acid, and α-ketoisovaleric acid. If any of these acceptors were to receive the 15 N-labeled amino group of phenylalanine, [^15^N]-alanine, [^15^N]-α-aminobutyric acid, [^15^N]-aspartic acid, [^15^N]-glutamic acid, [^15^N]-leucine, [^15^N]-methionine, or [^15^N]-valine would be formed accordingly.

**Figure 5:**
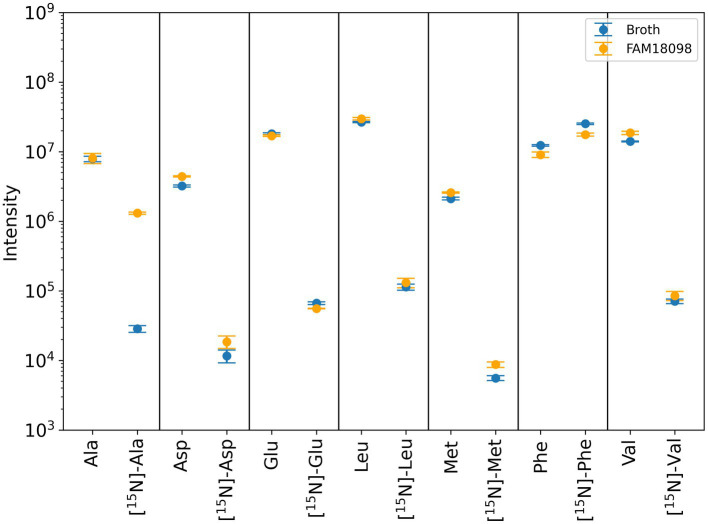
Determination of unlabeled and [^15^N]-labeled amino acids using mass spectrometry. Blue and orange dots represent the values determined in unfermented medium and medium fermented with *P. acidilactici* FAM 18098, respectively. Values represent the mean of three independent biological experiments (±standard deviation). Ala, alanine; Asp, aspartic acid; Glu, glutamic acid; Leu, leucine; Met, methionine; Phe, phenylalanine; Val, valine.

Of the amino acids mentioned, [^15^N]-α-aminobutyric acid was too low in concentration to be detected reliably in the medium or in the culture supernatant of FAM 18098. After fermentation with *P. acidilactici* FAM 18098, the levels of the unlabeled amino acids aspartic acid, methionine, and valine were found to be higher in the medium (Figure 5). No changes were observed for the unlabeled amino acids alanine, glutamic acid, leucine while phenylalanine was reduced. This pattern was also observed for the [^15^N]-labeled amino acids, except for alanine, which was clearly increased in the [^15^N]-labeled substance (Figure 5).

## 4. Discussion

Various biochemical changes take place in cheese during ripening. Among others, amino acids are released from the caseins during proteolysis, which are then further metabolized by bacteria. Transamination is one of the major steps for amino acid conversion. For example, Rijnen et al. (2003) showed that the knockout of transaminases in *Lactococcus lactis* impacts flavor formation in cheese.

*Pediococcus acidilactici* can also be found in cheese. Interestingly, this species forms α-aminobutyric acid and alanine (Eugster et al., 2019; Wenger et al., 2020). The enzymes involved in the formation of these substances are not yet known, and it is not inconceivable that a transaminase plays a role here. The analysis of the genome of *P. acidilactici* FAM 18098 (GenBank Assembly ASM980809v1) showed that this strain has six genes encoding aminotransferases (Wenger et al., 2020). For one of these six—which encodes the protein WP_159211138 and is named *aat* in this study—no function could be predicted from sequence analysis. Characterization of the gene product *in vitro* showed that the enzyme exhibits a broad substrate specificity by converting glutamic acid, leucine, methionine, phenylalanine, alanine, and α-aminobutyric acid (Wenger et al., 2020).

The broad substrate specificity of Aat did not allow us to assign it to a metabolic pathway. Therefore, a major objective of the present study was to disrupt the gene and investigate whether the knockout has an effect on α-aminobutyric acid and alanine biosynthesis.

For the knockout, a plasmid was constructed that replicates in *E. coli* and *P. acidilactici*. The procedure presented in this study has the advantage that a knockout plasmid can be produced from five PCR fragments. The disadvantage of the methodology is that the knockout leads to an integration of an antibiotic resistance gene, so that the modified strains can only be used for laboratory purposes.

### 4.1. Phenotypic traits of the knockout mutant

After confirming by PCR and whole genome sequencing that we had isolated a strain having lost the *aat* gene, the phenotypic characteristics of the wild-type strain were compared with the knockout strain. It was expected that the removal of an aminotransferase would affect amino acid and organic acid metabolism. Therefore, the composition of amino acids and organic acids was studied in the culture supernatants of the wild-type and knockout strains.

The comparison of the amino acid levels in the culture supernatants showed that the mutant and wild-type strains differed with respect to alanine, phenylalanine, and tyrosine. No differences were found for leucine, methionine, and α-aminobutyric acid —amino acids which Aat was able to convert *in vitro* (Wenger et al., 2020). It was concluded that Aat plays a role *in vivo*, mainly in the metabolism of alanine and the aromatic amino acids. This was confirmed when organic acid analysis revealed that the knockout mutant no longer synthesized PLA and HPLA (Figure 4).

**Figure 6:**
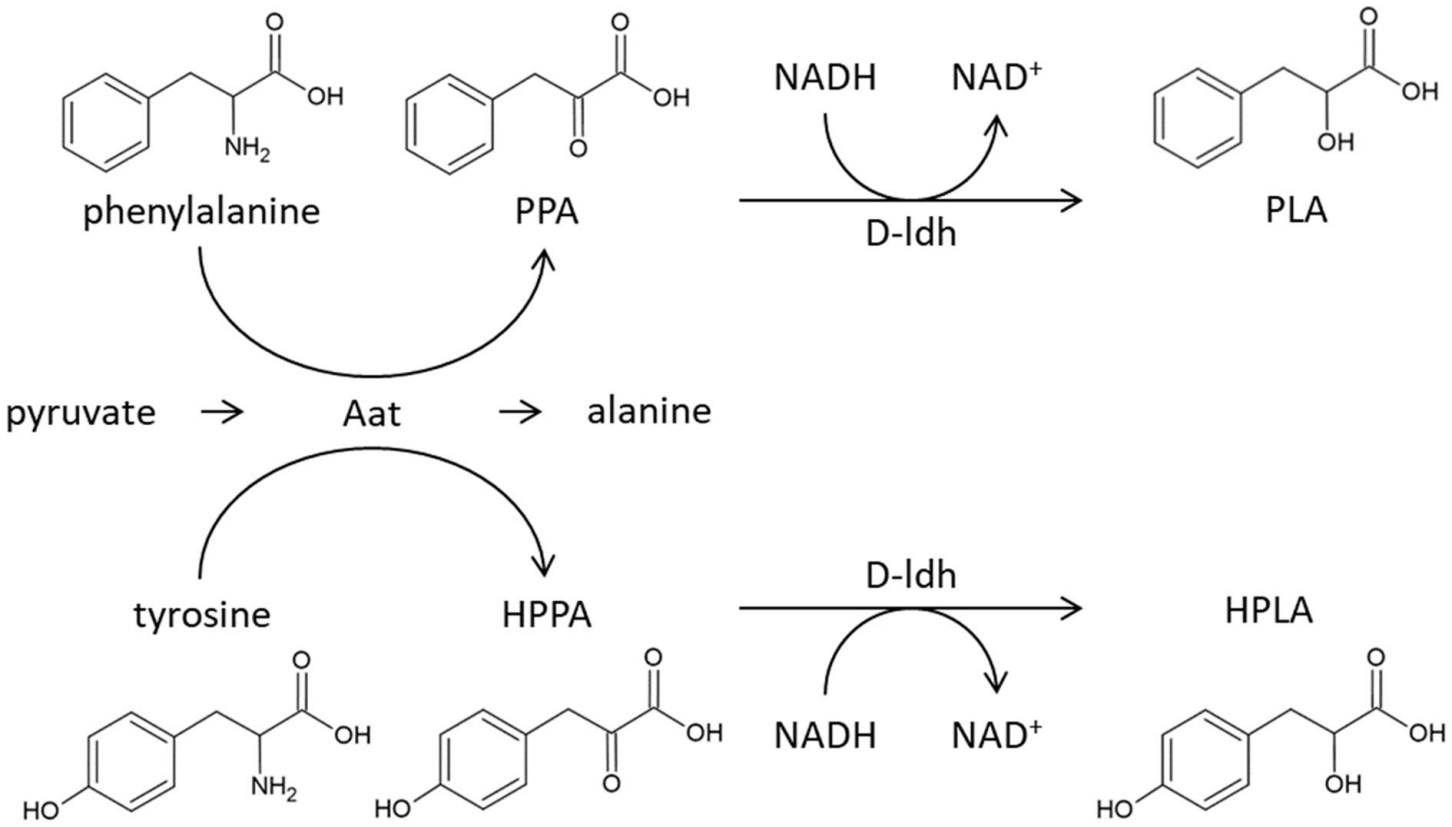
Proposed synthesis of 3-phenyllactic acid and 4-hydroxyphenyllactic acid in *Pediococcus acidilactici*. Firstly, phenylalanine and tyrosine are deaminated through the aminotransferase Aat (GenBank Protein WP_159211138), forming phenylpyruvic acid (PPA) and 4-hydroxyphenylpyruvic acid (HPPA), respectively. Then the keto acids are reduced by the D-lactate dehydrogenase (GenBank Protein WP_152689015) reaction, forming 3-phenyllactic acid (PLA) and 4-hydroxyphenyllactic acid (HPLA).

Rajanikar et al. (2021) reviewed in detail two biosynthetic pathways for PLA known in lactic acid bacteria. In the first case, named “*de novo* pathway,” glucose is the precursor for the biosynthesis of PLA and does not require the activity of an aminotransferase. In the second case, called the “core pathway,” phenylalanine is the precursor. Phenylalanine is thereby converted into phenylpyruvic acid by the action of an aminotransferase. Phenylpyruvic acid is subsequently reduced to PLA by a dehydrogenase (Figure 6).

Since PLA and HPLA production was no longer detected in the knockout mutant, it can be concluded that the two substances are mainly formed via the “core pathway” and the *aat* gene catalyzes the first step (Figure 6). This conclusion is supported by the study of Mu et al. (2012b), which showed that PLA and HPLA biosynthesis in *P. acidilactici* can be increased by the addition of phenylalanine and tyrosine, respectively.

The second step, the conversion of phenylpyruvic acid to PLA, is linked to a lactate dehydrogenase coding gene (*D-ldh*). This is based on the findings of Mu et al. (2012a), who showed that the heterologously expressed *D-ldh* reduced pyruvic acid to PLA.

There is increasing evidence showing that the genes of proteins that interact functionally are often located close to each other in the genome (Bhatt et al., 2018). However, this does not apply to *aat* and *D-ldh*, which are separated from each other by about 215,000 base pairs in the genome of FAM 18098. In addition to this fact, both enzymes exhibit broad substrate specificity. This suggests that the two enzymes not only play a role in PLA/HPLA biosynthesis but probably have other functions as well. This could also explain why PLA is often a by-product of cellular metabolism and modifications of the core pathway genes do not necessarily lead to high levels of PLA in biotechnological applications (Rajanikar et al., 2021).

### 4.2. Pyruvate acts as an amino-group acceptor

α-Ketoglutarate is the predominant amino group acceptor in many transamination reactions. The substance itself can be synthesized by glutamic acid dehydrogenase or isocitrate dehydrogenase. However, when the genome data of *P. acidilactici* FAM 18098 and other strains were analyzed, no genes encoding either of the two dehydrogenases were found (data not shown). This means that other amino group acceptors are involved in transamination reactions in *P. acidilactici*. When [^15^N] phenylalanine was used to analyze the transfer of nitrogen, the biosynthesis of [^15^N] alanine was observed, indicating that pyruvic acid is an amino group acceptor compound (Figure 5). Furthermore, it was observed that the [^15^N]-labeled phenylalanine was not completely catabolized. This is in line with findings from Mu et al. (2012b), who reported that *P. acidilactici* DSM 20284 does not convert all the available phenylalanine to PLA.

The present study shows that the production of knockout mutants is very helpful to elucidate metabolic pathways. Sequence analysis of Aat showed that it was annotated as a transaminase (EC 2.6.1.-), but depending on the search parameters and the database used, it was assigned to different EC numbers, such as EC 2.6.1.1 aspartate transaminase, EC 2.6.1.5 tyrosine transaminase, EC 2.6.1.9 histidinol-phosphate transaminase, or EC 2.6.1.57 aromatic-amino-acid transaminase. Analysis of the genetic context also did not help to assign the protein to a metabolic pathway (data not shown). When the enzyme was produced by heterologous expression, it exhibited broad substrate specificity (Wenger et al., 2020). With the knockout, it was then shown that *aat* plays a role in PLA/HPLA biosynthesis. As an outlook, comparative studies of cheese produced with the knockout and the wild-type strain could reveal whether other parameters, such as growth or flavor formation, are influenced by the newly characterized aminotransferase Aat.

## Data availability statement

The datasets presented in this study can be found in online repositories. The names of the repository/repositories and accession number(s) can be found in the article/Supplementary material.

## Author contributions

AW performed the experiments, analyzed the data, and wrote the first manuscript draft. RP assisted in the use and evaluation of the mass spectrometry analysis. AW, CB, and SI wrote the final manuscript. All authors provided intellectual input, reviewed the results, contributed to the discussion of the work and approved the final manuscript.

## Funding

This work was supported by Open access funding by Agroscope.

## Conflict of interest

The authors declare that the research was conducted in the absence of any commercial or financial relationships that could be construed as a potential conflict of interest.

## Publisher’s note

All claims expressed in this article are solely those of the authors and do not necessarily represent those of their affiliated organizations, or those of the publisher, the editors and the reviewers. Any product that may be evaluated in this article, or claim that may be made by its manufacturer, is not guaranteed or endorsed by the publisher.

## Supplementary material

The Supplementary material for this article can be found online at:

https://www.frontiersin.org/articles/10.3389/fmicb.2023.1150425/full#supplementary-material
