# Supplementary material for: The aminotransferase Aat initiates 3-phenyllactic acid biosynthesis in Pediococcus acidilactici
Source: Front Microbiol. 2023 Apr 28;14:1150425. doi: 10.3389/fmicb.2023.1150425 (PMC10175570; doi:10.3389/fmicb.2023.1150425)
Supplement: Supplementary file 1 [file Table_1.docx]

# Supplementary material

Supplementary Table 1: Retention times and masses used to detect and integrate peak areas of amino acids

| **Compound** | **Formula** | **Retention time [min]** | **[M+H]+** | **[M+H]+ [^15^N]** |
| --- | --- | --- | --- | --- |
| L-alanine | C_3_H_7_NO_2_ | 0.85 | 90.05495 | 91.052065 |
| α-aminobutyric acid | C_4_H_9_NO_2_ | 0.81 | 104.0706 | 105.067715 |
| L-aspartic acid | C_4_H_7_NO_4_ | 0.87 | 134.04478 | 135.041895 |
| L-glutamic acid | C_5_H_9_NO_4_ | 0.90 | 148.06043 | 149.057545 |
| L-leucine | C_6_H_13_NO_2_ | 1.75 | 132.10191 | 133.099025 |
| L-methionine | C_5_H_11_NO_2_S | 1.30 | 150.05833 | 151.055445 |
| L-phenylalanine | C_9_H_11_NO_2_ | 3.38 | 166.08626 | 167.083375 |
| L-valine | C_5_H_11_NO_2_ | 1.25 | 118.08626 | 119.083375 |
